# Enhancing the Antioxidant Activity of Technical Lignins by Combining Solvent Fractionation and Ionic‐Liquid Treatment

**DOI:** 10.1002/cssc.201901916

**Published:** 2019-09-24

**Authors:** Amel Majira, Blandine Godon, Laurence Foulon, Jacinta C. van der Putten, Laurent Cézard, Marina Thierry, Florian Pion, Anne Bado‐Nilles, Pascal Pandard, Thangavelu Jayabalan, Véronique Aguié‐Béghin, Paul‐Henri Ducrot, Catherine Lapierre, Guy Marlair, Richard J. A. Gosselink, Stephanie Baumberger, Betty Cottyn

**Affiliations:** ^1^ Institut Jean-Pierre Bourgin, INRA, AgroParisTech, CNRS Université Paris-Saclay 78000 Versailles France; ^2^ FARE Laboratory, Fractionnement des AgroRessources et Environnement INRA Université de Reims Champagne Ardenne 51100 Reims France; ^3^ Wageningen Food and Biobased Research 6708 WG Wageningen The Netherlands; ^4^ Institut National de l'Environnement Industriel et des Risques (INERIS) Parc Technologique Alata, BP2 60550 Verneuil-en-Halatte France

**Keywords:** antioxidants, biorefinery, green chemistry, ionic liquids, lignins

## Abstract

A grass soda technical lignin (PB1000) underwent a process combining solvent fractionation and treatment with an ionic liquid (IL), and a comprehensive investigation of the structural modifications was performed by using high‐performance size‐exclusion chromatography, ^31^P NMR spectroscopy, thioacidolysis, and GC–MS. Three fractions with distinct reactivity were recovered from successive ethyl acetate (EA), butanone, and methanol extractions. In parallel, a fraction deprived of EA extractives was obtained. The samples were treated with methyl imidazolium bromide ([HMIM]Br) by using either conventional heating or microwave irradiation. The treatment allowed us to solubilize 28 % of the EA‐insoluble fraction and yielded additional free phenols in all the fractions, as a consequence of depolymerization and demethylation. The gain of the combined process in terms of antioxidant properties was demonstrated through 2,2‐diphenyl‐1‐picrylhydrazyl (DPPH^.^) radical‐scavenging tests. Integrating further IL safety‐related data and environmental considerations, this study paves the way for the sustainable production of phenolic oligomers competing with commercial antioxidants.

## Graphical abstract

**Technical lignin refinement**: A cascade biorefinery process is presented that enables upgrading of technical lignins and sustainable production of phenolic oligomers of interest as substitutes for commercial antioxidants.

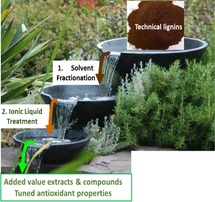

## Introduction

Industry is increasingly demanding of biobased phenolic compounds, to be used as building blocks for polymer synthesis or valued for their antiradical activity, especially in the field of polymers, materials, and cosmetics. Phenolics derived from plant biomass are in particular potential alternatives to synthetic commercial antioxidants such as Bisphenol A^1^ and *tert*‐butylated hydroxytoluene (BHT).^2^ Among them, ferulic acid is already known for its potential for various applications,^3^ but its low availability from plant sources might hinder its industrial development. As polymers of phenyl propanoids representing up to 30 % of the plant biomass, lignins represent one of the major potential sources of biobased phenolics.^4^ Moreover, the valorization of technical lignins generated as industrial byproducts would increase the sustainability of lignocellulose‐based biorefineries.^5^

**Figure 1:**
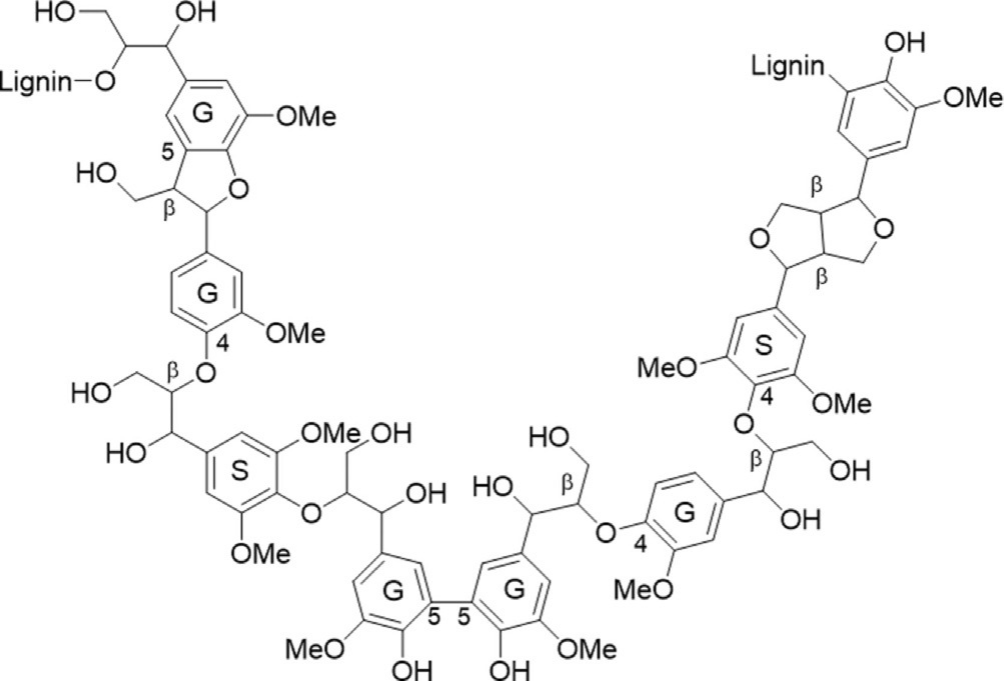
Chemical structure of lignin showing the G and S phenylpropane units linked together by inter‐unit labile aryl–alkyl ether bonds (β‐O‐4) and resistant bonds (5‐5, β‐5, β‐β).^10^

Various strategies driven by green chemistry principles have been designed to convert technical lignins into functional molecules or assemblies while mitigating their variability processing from botanical origin and transformations during industrial treatments.^6^ These strategies include fractionation and depolymerization. Fractionation can be performed by ultrafiltration, potentially directly integrated into the pulping process,^7^ or by solvent extraction, which is advantageously performed at ambient temperature and pressure and does not require specific equipment.^8^ In contrast, among the depolymerization strategies (thermochemical, biological, or chemocatalytic),^9^ the recently reported implementation of methyl imidazolium bromide ([HMIM]Br) ionic liquid (IL) provides a way to produce lignin oligomers with increased free phenol content (PhOH) and decreased polymerization degree, as a consequence of the selective cleavage of aryl–alkyl ether bonds (Figure 1).^10^ In particular, this was shown to generate new phenol groups from the methoxy groups of lignin syringyl and guaiacyl units. Such phenolic oligomers have great potential as antioxidant additives for the formulation of materials because they are likely to combine miscibility in the polymer matrix and limited migration towards the environment owing to their oligomeric structure.^11^, ^12^ Despite this potential, the process has, until now, only been applied to lignin models, and the antioxidant properties of the oligomers have not been assessed yet. The advantages of using [HMIM]Br for the transformation of lignins are foreseen to potentially ensure the homogeneity of the reaction medium, to avoid the use of additional chemical reagents, and to perform the reaction in mild conditions (*T*<110 °C, *t*<40 min) compared with most lignin catalytic conversion processes. Moreover, in this context of use, the IL can be recycled. All these advantages also render the process attractive from an environmental point of view. However, the sustainability assessment of the process requires further health and safety information on [HMIM]Br, which has not been provided so far. The present paper aims at assessing the possibility of transferring the newly developed IL process^10^ to technical lignin fractions. The objective was to recover antioxidant extracts soluble in ethyl acetate (EA), one of the conventional solvents recommended for their relatively limited health hazard and environmental footprint as assessed from the Health and Safety Executive (HSE) criteria compiled from various sources.^13^ EA was selected here to separate extractives from the IL/water layer. Because alkali grass lignin Protobind 1000 (PB1000) from GreenValue LLC is available at an industrial scale and is already known for its antioxidant properties,^14^ it was selected as the starting material for this study. This lignin was subjected to a three‐step semi‐continuous solvent fractionation process to recover three structurally distinct soluble fractions (F1–F3). In parallel, the same lignin sample was submitted to an extensive EA washing to recover an EA‐insoluble residue (F4) and validate the possibility of recovering soluble compounds through chemical treatment of this fraction. PB1000 and the four fractions were submitted to the previously optimized IL treatment by using microwave irradiation (MW) or conventional heating (CH) as heating processes. The advantage of MW is the short reaction time (10 s), whereas CH was used to generate more severe modifications relevant to investigation of the structure–properties relationships. The phenolic monomers and oligomers were extracted from the reaction media with EA, and the ethyl acetate extracts (EAE) were analyzed by chromatographic and spectroscopic methods to assess the efficiency of the conversion, elucidate mechanisms, and identify molecules of interest for further developments. The antioxidant properties of PB1000 and the EAE as well as those of the insoluble residues recovered from the most drastic process (CH, 40 min) were assessed through 2,2‐diphenyl‐1‐picrylhydrazyl (DPPH^.^) radical‐scavenging tests. The half‐max‐effective concentration EC_50_ was used as the criterion to compare the performance of the different samples. The study demonstrates the technical advantage of implementing the [HMIM]Br‐based treatment in a cascading approach combining fractionation and depolymerization. In addition, sustainability considerations of the process are further discussed according to safety data obtained for the IL.

## Results and Discussion

### Recovery of different PB1000 fractions and their contrasted reactivity towards [HMIM]Br treatments

#### PB1000 fractionation

**Figure 2:**
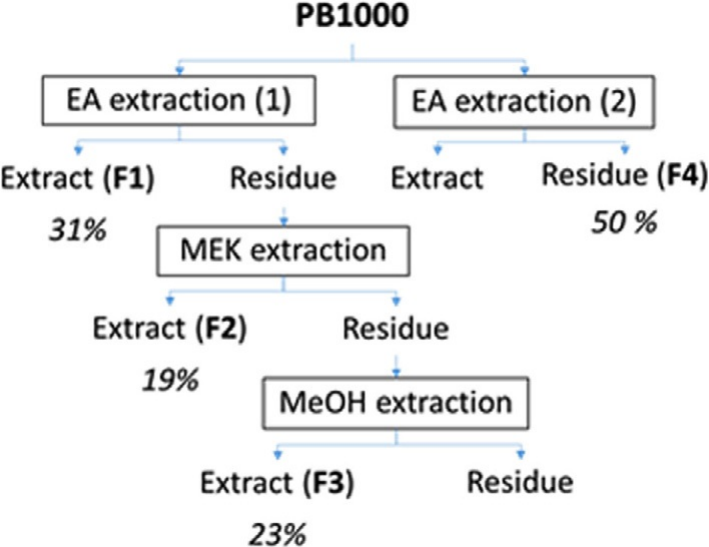
Fractionation scheme of PB1000 by sequential extraction with EA, MEK, and MeOH, and recovery yields [wt %/PB1000] of the fractions (F1–F4).

PB1000 was fractionated at a semi‐pilot scale (1 kg dry powder) through a semi‐continuous process intended for future industrial development.^8^ This process consisted of a three‐step sequential extraction with solvents of increasing polarity (Figure 2) and yielded 31 wt % for the EA‐soluble fraction F1, 19 wt % for the butanone‐ (MEK)‐soluble fraction F2, and 23 % for the MeOH‐soluble fraction F3. Because F2 and F3 contained residual EA‐soluble compounds (37 and 7 %, respectively), PB1000 was also submitted, at lab scale [g], to a drastic extensive washing with EA yielding an EA‐insoluble fraction F4 (50 % of the weight of the starting PB1000) subsequently used only to validate the depolymerization process.

One main characteristic of this set of four fractions was the increasing proportion of lignin inter‐unit aryl–alkyl β‐O‐4 linkages from F1 to F4, as reflected by the increasing thioacidolysis yields (Table 1).^15^ Because our previous study performed on dioxan‐isolated model lignins showed the β‐O‐4 linkages to be the most privileged target of the IL treatment allowing partial depolymerization,^10^ these fractions were thus expected to show increasing reactivity towards the IL treatment.

**Table 1:** Thioacidolysis yield of PB1000 and its fractions.

Samples	Thioacidolysis yield [μmol g^−1^]
PB1000	83
F1	32
F2	96
F3	166
F4	197

**Figure 3:**
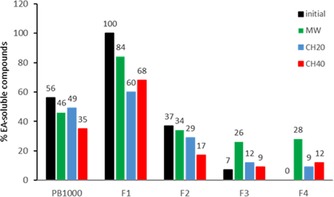
EA‐soluble compounds proportion [wt %] of the initial samples (PB1000 and its fractions F1–F4) and the reaction products recovered upon [HMIM]Br treatments with MW, CH20, and CH40. Values after treatments are calculated based on EAE and EA extraction residues (EAR) recovery yields.

To determine the influence of structural variations on the efficiency of the depolymerization process, PB1000 and its four derived fractions were subjected to heating treatments in [HMIM]Br, either under MW (10 s, 110 °C) or under CH [110 °C, 20 (CH20) or 40 min (CH40)]. In line with the objective of the treatment to recover EA‐soluble functional extracts, the efficiency of the treatments was assessed on the basis of the recovery yields of the EAE after treatment and the characteristics of the extracts, selecting average molar masses and PhOH as the major criteria for further applications (Table 2). Moreover, to estimate the solubility gain, the proportion of EA‐soluble compounds with respect to the total products recovered was calculated and compared with the EA solubility of the initial sample (Figure 3).

**Table 2:** Yields and characteristics of the EAE recovered after treatment by [HMIM]Br with MW, CH20, and CH40 compared to the initial sample PB1000 and its fractions F1–F4.

Samples	EAE yield^[a]^ [%]		PhOH^[b]^	*M*_n_^[c]^	*M*_w_^[c]^	PD
	/SF	/PB1000	[mmol g^−1^]	[g mol^−1^]	[g mol^−1^]	[d]
**PB1000**						
initial	56	56	2.68	1015	1260	1.2
MW	36	36	4.55	580	814	1.4
CH20	45	45	3.95	606	843	1.4
CH40	29	29	4.22	802	936	1.2
						
**F1**						
initial	100	31	3.88	896	1089	1.2
MW	72	22	4.43	805	1190	1.5
CH20	59	18	5.45	664	882	1.3
CH40	59	18	6.62	680	876	1.3
						
**F2**						
initial	37	7	2.76	914	1085	1.4
MW	28	5	4.14	978	1407	1.4
CH20	26	5	5.74	700	935	1.3
CH40	16	3	8.27	676	872	1.3
						
**F3**						
initial	7	2	1.18	961	1212	1.3
MW	21	5	2.78	851	1140	1.3
CH20	9	2	7.26	752	945	1.3
CH40	7	2	11.94	709	937	1.3
						
**F4**						
initial	0	0	1.59	1165	1856	1.6
MW	15	8	6.25	865	1144	1.3
CH20	5	2	3.96	867	1153	1.3
CH40	7	3	4.64	971	1282	1.3

[a] EAE recovery yields expressed with respect to the mass of sample treated by [HMIM]Br (/SF) and to the mass of PB1000 submitted to fractionation before treatment (/PB1000). [b] Determined by ^31^P NMR spectroscopy after phosphorylation in pyridine/deuterated chloroform of the whole initial samples and of the EAE recovered after IL treatment. [c] High‐performance size‐exclusion chromatography (HPSEC) determination after dissolution/filtration in THF of the EAE recovered before and after IL treatment (values given for PB1000 and F4 before treatment correspond to the whole samples). [d] Polydispersity.

#### Effect of the [HMIM]Br treatment on the recovery of EAE

As a consequence of the fractionation process, the four fractions exhibited initial contrasted solubility in EA: F1 was 100 % EA‐soluble, whereas F4 was poorly soluble, and F2 and F3 only partially (37 and 7 %, respectively). The [HMIM]Br treatments induced a decrease in the proportion of EA‐soluble compounds of all samples, except for F3 and F4, which conversely exhibited an increase. The highest effect of the [HMIM]Br treatment was observed for F4 under the MW conditions. Indeed, 15 % of this insoluble fraction could be solubilized, and 28 % of the products formed was soluble. The EAE represented 8 % of PB1000. Three‐fold and two‐fold lower solubilities were observed with CH20 and CH40 conditions, respectively.

In contrast, the treatment of the totally soluble fraction F1 led to the decrease of the solubility with production of insoluble material (16–40 % depending on the treatment). The maximum solubility decrease was observed with the CH conditions, indicating that these conditions potentially favored recondensation reactions of the EA‐soluble compounds owing to the longer reaction time. In the case of the only partially EA‐soluble fractions F2 and F3, two contrasting behaviors were observed: the F2 behavior was similar to F1 with a solubility decrease enhanced in CH conditions, and the F3 behavior was similar to F4 with solubility increase (up to 19 % solubility increase) under MW conditions. The EAE recovered from F3 accounted for 21 % of the fraction and 5 % of PB1000. The treatment of PB1000 without any previous fractionation led to an intermediate behavior with a solubility decrease observed only in the most severe conditions, CH40. In conclusion, the [HMIM]Br treatment could produce EA‐soluble compounds from fractions showing poor initial EA solubility, and the presence of EA‐soluble compounds in the initial sample led to an apparent decrease in EA solubility, which was most probably owing to recondensation reactions favored by CH conditions.

Besides initial EA solubility, the fraction‐dependent effect of [HMIM]Br treatment on the amount of EAE recovered could be related to the structural differences of the fractions in terms of β‐O‐4 bond content. Indeed, in the case of F1 and F2, which exhibit the lowest amount of β‐O‐4 bonds according to thioacidolysis yields, the proportion of EA‐soluble compounds in the reaction mixtures after treatment decreased, whereas in the case of F3 and mainly F4, this proportion significantly increased. The results are in agreement with our previously reported statements^10^ and suggest that β‐O‐4 linkages are effectively the most privileged targets of the IL treatment. This also demonstrates that the efficiency of the depolymerization process is able to counterbalance the effect of recondensation reactions occurring during the treatment, and that may be particularly important in the case of long reaction times (CH compared with MW).

In all cases, and whatever the treatment, the yield of the reaction was good but never reached 100 % owing to losses of compounds, mainly volatiles (not identified nor quantified), during the reaction and soluble and insoluble compounds during the separation process (precipitation of the products and removal of the water/IL layer).

#### Molar mass distribution of the EAE

Whatever the sample, the weight‐average molar mass (*M*_w_) of the EAE, before or after treatment, did not exceed 2000 g mol^−1^, indicating that the EAE were essentially composed of oligomers (less than ten phenylpropane units). Nevertheless, some variations in molar distribution appeared between the samples upon treatments. Increasing EAE average molar masses was observed from F1 to F3, in agreement with the use of solvent with increasing polarities for the PB1000 fractionation process.^16^, ^17^ The effect of the [HMIM]Br treatment on the molar masses depended on the fraction and conditions used. In the case of F3, all treatments induced a decrease in average molar masses with an increasing effect from MW to CH20 and CH40. For F1 and F2, a decrease in the molar mass was only observed in CH conditions, and the use of MW by contrast led to a slight increase. In the case of F4, the EAE produced by the treatments exhibited molar masses 1.4‐ to 1.6‐fold lower than that of the initial F4 insoluble fraction, and of the same range as the non‐modified EAE of the other fractions. A slight increase was observed in the CH conditions. This result supported the hypothesis that recondensation reactions were favored by the CH conditions.

#### Evidence of demethylation and depolymerization upon [HMIM]Br treatment

GC–MS analysis was performed to investigate the phenolic monomers present in the EAE (Supporting Information, S1). It revealed the presence of a mixture of compounds (phenolic acids, ketones, and aldehydes accounting in total for 1.2 % of PB1000) in F1, the absence of monomeric compounds in F2 and F3 before treatment, and the formation of new compounds for all fractions after treatment. The chromatograms of the EAE recovered after treatment revealed the presence of phenolic monomers diagnostic of demethylation (all fractions) and/or depolymerization (F2 and F3). In F1, the treatment led to the total conversion of acetosyringone, the major EAE phenolic monomer before treatment, into its once‐ and twice‐demethylated counterparts [1‐(3,4‐dihydroxy‐5‐methoxyphenyl)‐ethan‐1‐one **C** and 1‐(3,4,5‐trihydroxyphenyl)‐ethan‐1‐one **D**] and to the disappearance of all other phenolic extractives, most probably involved in recondensation reactions. In F2 and F3, the treatment led to the production of two ketones **A** and **B** previously identified as acidolysis ketones from experiments on lignin models.^10^ In F2, only the demethylated form **B** was detected whereas in F3 both were formed. The analysis of the thioacidolysis products also indicated that demethylation took place within lignin units linked through β‐O‐4 bonds (Supporting Information, S2). Indeed, catechol, 5‐hydroxyguaiacyl, and 3,4,5‐trihydroxyphenyl thioethylated derivatives were detected after thioacidolysis of all treated samples. The proportion of these demethylated units was higher in the CH‐treated samples, with an increased effect after 40 min of CH. An important feature arising from these results is that demethylation and depolymerization through the cleavage of β‐O‐4 bonds seem to be concomitant but independent processes. Both the demethylation and depolymerization reactions diagnosed herein allow the appearance of new phenolic groups initially involved in ether bonds and could thus account for the increase of PhOH.

#### PhOH functionality gained by IL treatment combined with EA extraction

To assess the functionality gained through IL treatment combined with subsequent extraction, the PhOH content of the EAE after treatment were compared with the initial PhOH content of the samples. As shown by the higher PhOH content of F1 (the PB1000 EA‐soluble fraction) compared with PB1000, EA extraction alone provided a way to recover compounds with higher functionality. However, this functionality was even higher when the IL treatment was applied before recovery of the EAE (3.95–11.94 versus 3.88 mmol g^−1^), except for F3‐MW (2.78 mmol g^−1^). Moreover, whatever the sample and the conditions, the [HMIM]Br treatment led to an increase of PhOH compared with the starting sample. This effect increased from F1 to F3, for which a maximum ten‐fold increase was obtained (Table 2). In the case of F3, the increase in PhOH was concomitant to the decrease in average molar masses, suggesting that the treatment induced the cleavage of ether bonds with subsequent release of phenol groups, as previously demonstrated on lignin models.^10^

**Figure 4:**
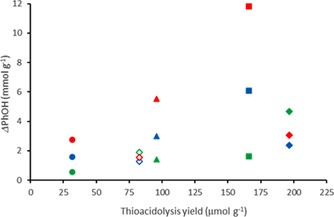
Enrichment in PhOH upon MW (green), CH20 (blue), and CH40 (red) [HMIM]Br treatments (difference between the EAE PhOH content after treatment and the initial PhOH content of the sample) as a function of the thioacidolysis yield of the initial samples PB1000 (◊), and its fractions F1 (•, F2 (▴), F3 (▪), and F4 (⧫).

The PhOH enrichment (ΔPhOH, mmol g^−1^) through IL treatment and subsequent EA extraction increased with the thioacidolysis yield of the initial fraction, whatever the treatment (Figure 4). This was consistent with the major contribution of depolymerization reactions through β‐O‐4 bond cleavage to form new phenol groups. However, PB1000 and its residual F4 fraction did not followed the same tendency as the other three fractions. In particular, the *Δ*PhOH upon CH treatments was lower than expected from the behavior of these fractions. This suggested that a higher proportion of phenols formed by depolymerization was present in the EA‐insoluble residues compared with the other fractions. Moreover, the weak differences in terms of mass distribution, and in fact the unclear correlation between thioacidolysis yield of the starting fraction and the *M*_w_ of the obtained EAE, once more indicated that in the case of fractions for which the depolymerization process could be highly efficient (F3 or F4), recondensation reactions also occurred. Interestingly, these recondensations reactions seemed not to impact the global PhOH content. This could be also proof of a synergistic effect between both demethylation and depolymerization processes. In all cases, the efficiency of the treatment conditions in terms of phenols production was increased from MW to CH20 and CH40. As a major feature, the [HMIM]Br treatment was shown to induce an increase of PhOH content, which may be the consequence of both depolymerization and transformation of methoxy groups into phenol groups.

To assess the effect of the PhOH increase on the antioxidant properties, the EAE showing the highest PhOH content (CH40 samples) were selected for the further radical‐scavenging tests. The corresponding EA‐insoluble residues were also tested in view of the potential use of all fractions of PB1000.

### Interest in the products of fractionation and [HMIM]Br treatment as potential antioxidants

#### Antioxidant properties (AOP) of the phenolic monomers compared with reference antioxidants

**Figure 5:**
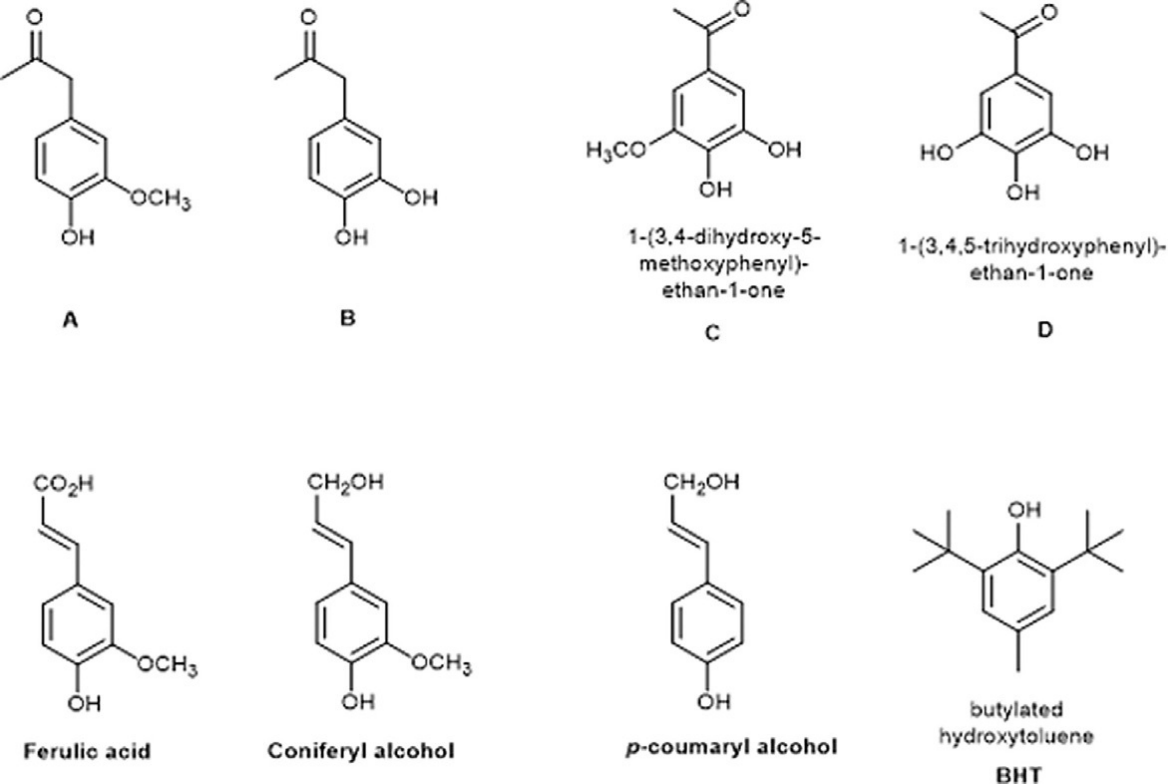
Structures of phenolic compounds compared for their radical‐scavenging activity: (**A**–**D**) compounds detected by GC–MS in the EAE after IL treatment of PB1000 fractions, lignin model compounds as monolignols (ferulic acid, coniferyl alcohol, *p*‐coumaryl alcohol), and a commercial antioxidant (BHT).

The main phenolic monomers detected in the EAE after treatment, namely acidolysis ketones (compounds **A** and **B**, Figure 5) and acetosyringone demethylated once or twice (compounds **C** and **D**, Figure 5), were tested for their DPPH^.^ radical‐scavenging capacity according to a test previously used to compare the AOP of lignin models and technical lignins.^14^ EC_50_ (concentration of tested sample necessary to reduce 50 % of the radicals) was used for this comparison.

**Figure 6:**
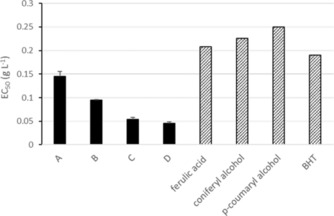
DPPH^.^ radical‐scavenging capacity of phenolic compounds produced by IL treatment of PB1000 fractions (compounds **A**–**D**) compared with monolignols and a commercial synthetic antioxidant (BHT). Error <1 %.

**A**, **B**, **C**, and **D** showed higher AOP (EC_50_<0.2 g L^−1^) than the other lignin model compounds tested (ferulic acid, coniferyl alcohol, and *p*‐coumaryl alcohol; Figure 6). The lowest EC_50_ was reached with the twice‐demethylated acetosyringone (compound **D**)—four times lower than that of ferulic acid (0.21 g L^−1^), which is a reference for natural antioxidants. The radical‐scavenging capacity of lignins, and phenolics in general, relies on the ability of phenols to trap free radicals in the medium after the loss of a proton^18^ followed by the stabilization of this radical by mesomerism. The best performance of compounds **C** and **D** is consistent with the presence of highly electron‐withdrawing groups conjugated with the aromatic ring and with the presence of several phenols carried by adjacent carbons.^19^

Interestingly, all tested compounds were competitive with a commercial antioxidant such as BHT (EC_50_=0.19 g L^−1^). Thus, these compounds might be advantageously purified or synthesized and used as antioxidants for commercial applications. However, to avoid costly purification steps and to profit from the possible synergies between phenolic compounds, the AOP of the mixtures of products recovered from the treatments [EAE and EA extraction residues (EAR)] was considered.

#### AOP of EAE and EAR compared with PB1000 and its fractions

All fractions (F1–F3) and the products recovered from the CH40 IL treatment of these fractions exhibited DPPH^.^ radical‐scavenging capacities similar to or higher than that of the PB1000 reference sample, according to the EC_50_ data (Table 3). Concerning the untreated fractions, the results are in accordance with previous studies on the fractionation of other technical lignins (organosolv lignin BIOLIGNIN and LignoBoost Kraft lignins), showing the generally higher radical‐scavenging activity of the EA‐soluble fraction compared with the other ones.^20^ The IL treatment of the fractions led to the production of EAE with enhanced radical‐scavenging activity compared with PB1000 and the fraction considered. This enhancement could be explained by the increased PhOH content after treatment and the presence of the highly antioxidant phenolic monomers in EAE. In addition, EAR also exhibited interestingly higher activity than their corresponding starting fractions, except for F1. In the case of F3, the AOP of the insoluble residue was even twice that of the EAE, which confirmed that some new‐formed phenol groups were carried by some compounds of higher molar mass insoluble in EA after treatment. According to the EC_50_, F2 appeared as the most interesting fraction with respect to the production of antioxidants through IL treatment because the radical‐scavenging activity of both the EA‐soluble and ‐insoluble fractions after treatment were the best.

**Table 3:** DPPH^.^ radical‐scavenging capacity (EC_50_) of PB1000, its fractions (F1–F3), and their reaction products (EAE and EAR) recovered upon [HMIM]Br IL treatment with CH40.

Samples	EC_50_ [g L^−1^]		
	Untreated	EAE	EAR
PB1000	0.40±0.01	–	–
F1	0.27±0.03	0.11±0.00	0.42±0.08
F2	0.27±0.01	0.11±0.00	0.15±0.01
F3	0.38±0.03	0.33±0.01	0.17±0.01

#### Benefit of combining fractionation and IL treatment

**Figure 7:**
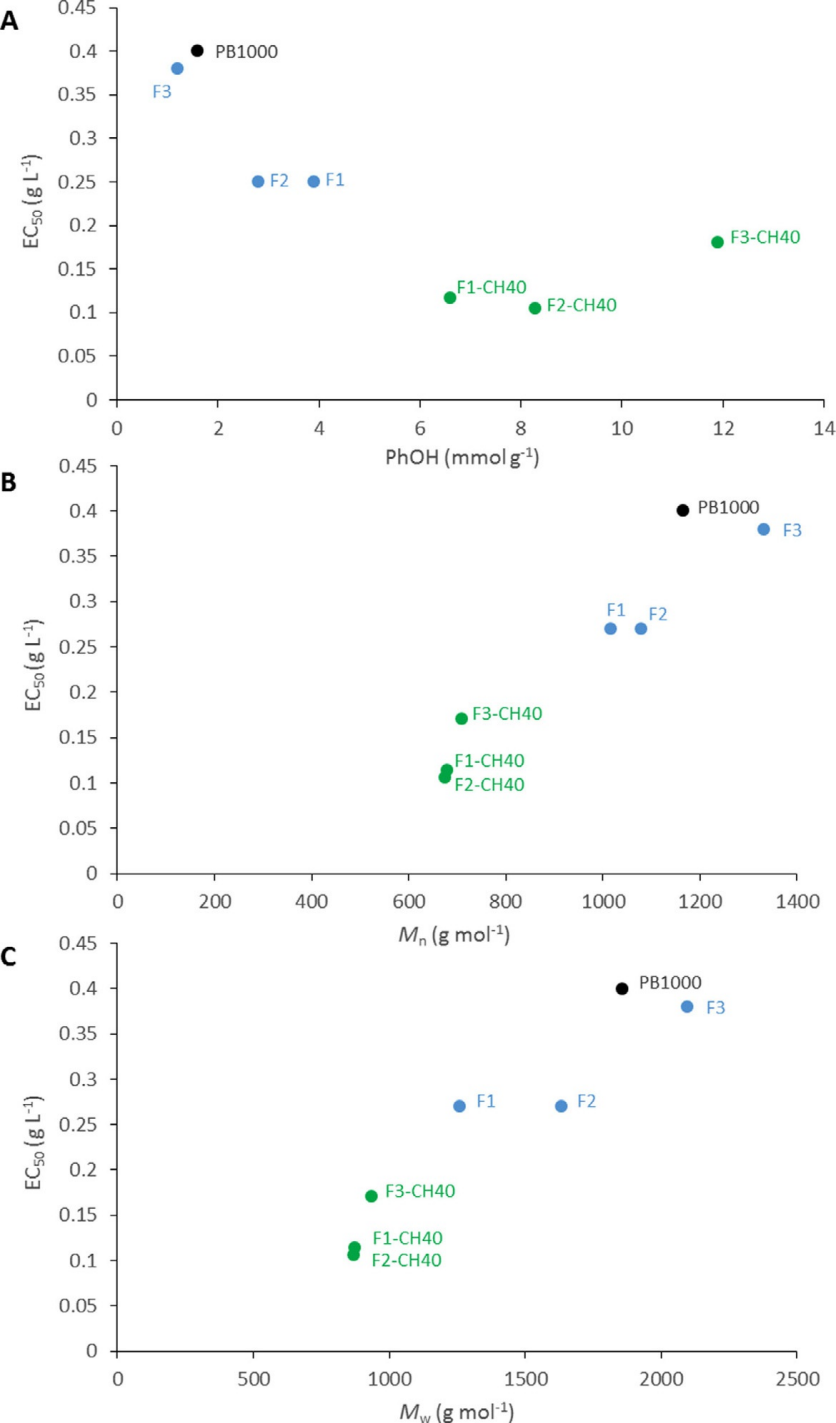
Mapping of PB1000 and its products according to (A) EC_50_ and PhOH content, (B) EC_50_ and *M*_n_, and (C) EC_50_ and *M*_w_: starting PB1000 sample (black), PB1000 fractions (blue), EAE recovered after [HMIM]Br treatment with CH40 (green).

Owing to PB1000 fractionation and the subsequent IL treatment of the fractions, a set of functional antioxidant products with distinct characteristics was generated. The mapping of the different samples according to their antioxidant property (EC_50_), molar mass distribution (*M*_n_, *M*_w_) and phenolic content (Figure 7) highlights that the EAE recovered after IL treatment of the fractions combined several advantages: lower molar masses (*M*_n_≈700 g mol^−1^), higher PhOH content (6–12 mmol g^−1^), and higher radical‐scavenging activity (EC_50_=0.10–0.17 g L^−1^).

Moreover, it shows that the molar mass differences between the fractions were levelled by the IL treatment. These characteristics make them good candidates as antioxidant ingredients for the formulation of plastics or cosmetics or for the synthesis of new biobased polymers and multifunctional molecules. Based on these results, an integrated process could be proposed, and its sustainability was discussed.

### Towards a sustainable cascade integrated process

Taken together, the results showed that IL treatment could be advantageously applied to lignin fractions for different purposes: production of competitive antioxidant extracts, increase of lignin free phenol content, and partial dissolution of insoluble residues. In all cases, the demethylation and depolymerization induced by the treatment is beneficial. To design a cascade approach and preliminarily assess its sustainability, it is necessary to consider the technical performances of the products along with the recovery yields and safety aspects.

#### Recovery yields and technical gain

**Figure 8:**
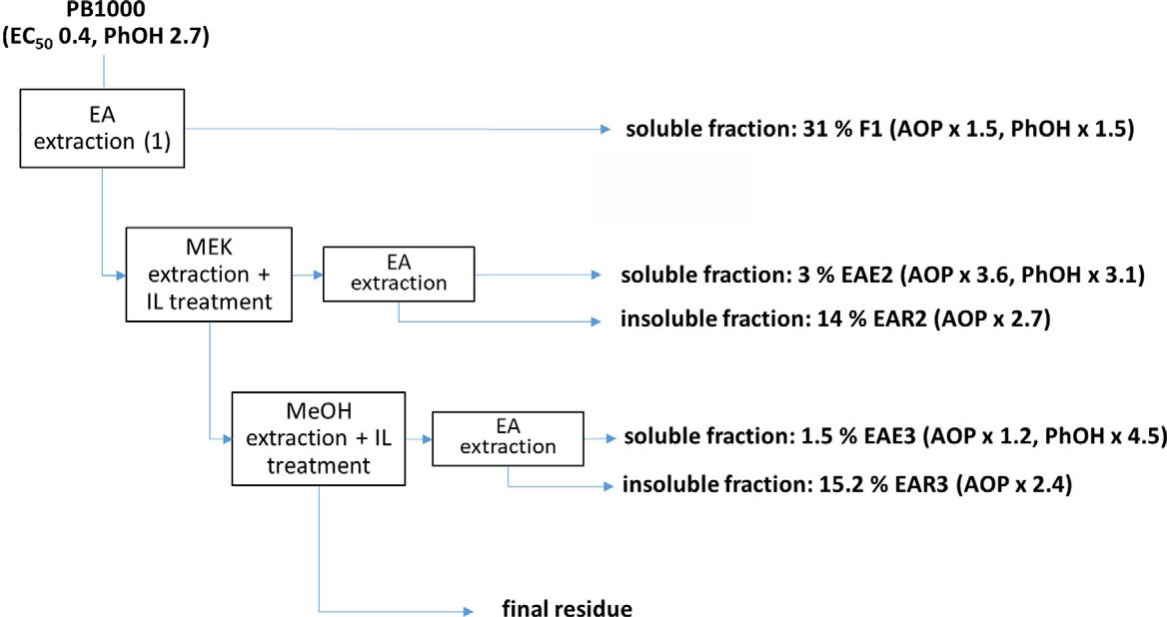
Scheme of a process proposal yielding 35.5 % of EA‐soluble products (F1, EAE2, EA3) and 29.2 % EA‐insoluble products (EAR2, EAR3) showing a gain in terms of antioxidant property (AOP=1/EC_50_) and free PhOH content compared with the starting material.

The process proposed in Figure 8 allows the production of a total of 67.7 % of products with enhanced antioxidant activity compared with PB1000, including 35.5 % EA‐soluble oligomers enriched in PhOH. To optimize the gain in AOP and PhOH, the IL treatment conditions selected are the most drastic ones (CH40), which on the contrary do not favor the formation of soluble material. However, because the insoluble products exhibit high antioxidant properties, they could be advantageously incorporated directly as fillers in plastics or functionalized by grafting of the PhOH. In the first step of the process, a functional fraction is directly obtained from PB1000 by EA extraction. In the second step, the residue is submitted to a MEK extraction combined with IL treatment of the extract to recover both EA‐soluble and ‐insoluble highly antioxidant products. In the last stage, the MEK extraction residue undergoes a MeOH extraction combined with IL treatment to recover EA‐soluble oligomers highly enriched in phenol groups together with antioxidant insoluble compounds. Owing to its lowest content in lower‐molar‐mass phenolic extractives, the final residue of the process might find applications, for instance, as a filler in materials.^21^

#### Safety‐ and environment‐related aspects and further sustainability considerations

Sustainability assessment of the developed value chain was further examined for physicochemical hazards and environmental impacts potentially arising from the key chemicals involved. With regard to the fire hazard, the two main critical points of the process in terms of safety are the use of organic solvents (EA, MEK, and MeOH) for the extraction steps and the use of a new ionic liquid ([HMIM]Br). Of course, the well‐established flammability of the mentioned solvents has to be handled from supply to process end‐use, which leads in this particular context to limited issues according to mild operations conditions. Despite their flammability properties, such materials remain still recommendable as extraction solvents, with regard to HSE criteria taken into consideration with some prioritizing rules.^22^ In particular, these substances are not mentioned in any list of the EU REACH regulation designating substances of particular concern owing to their adverse health or environmental impacts. A summary of results pertaining to physical hazards potentially associated to the use of [HMIM]Br is provided in Table 4.

**Table 4:** Data summary pertaining to fire and corrosivity to metal hazards.

Hazard	EA	MeOH	MEK	[HMIM]Br
ignitability	easy	easy	easy	very difficult
heat release rate	medium	moderate	large	very low
fire retardancy	none	none	none	very significant
fire‐induced toxicity	CO_*x*_ emissions in fire	CO_*x*_ emissions in fire	CO_*x*_ emissions in fire	CO_*x*_, HCN from unexpected fire conditions
corrosivity to metals (C and stainless steel)	n.d.	n.d.	n.d.	significant for neat IL and IL with 10 % added water

n.d.=not determined.

Moreover, EA and MeOH could potentially be substituted with biobased recommended alternatives, bio‐ethyl lactate and bioethanol, respectively (the latter is nowadays totally biosourced). MEK, if not so easily substituted, and despite some toxicity hazard, might at least be produced from biomass in the future, in a cost‐effective way as an intermediate to biobutanol.^23^

Although often considered as green solvents, ILs remain controversial for safety issues^24^ and chiefly require a case‐by‐case analysis in the context of use because safety assessments are not based only on intrinsic properties but essentially on apparatus and testing procedure‐dependent properties (flash point, thermal stability, metal corrosivity).^25^ A preliminary assessment of the [HMIM]Br safety profile has been performed and is provided herein (with technical details in the Supporting Information, S3), integrating both physicochemical hazards (fire, corrosivity to metals) and eco‐toxicological properties of the IL, in line with REACH regulation safety data needed for future registration. This study also provides new insights regarding [HMIM]Br thermal stability and fire behavior, showing in particular a remarkable resistance to ignition and a flame retardancy property. A first‐order evaluation of the corrosivity potential of the neat IL has indicated that further investigation on the matter will be required for the appropriate selection of the reactor material owing to practical conditions for use of the IL.

Regarding the environmental properties of the IL,^26^ the selected test battery includes the tests required by annex VII of the REACH regulation (substance manufactured or imported into the European Union in quantities between 1 and 10 tons per year) and immunotoxicity tests on the three‐spined stickleback (*Gasterosteus aculeatus*). The results (Table 5) showed that [HMIM]Br has low the toxicity for *Daphnia magna* and *Pseudokirchneriella subcapitata* (EC_50_>100 mg L^−1^). No biodegradation was observed in the manometric respirometry test, leading us to conclude that [HMIM]Br is not rapidly biodegradable. These results are consistent with those already obtained on compounds of the imidazolium family, which demonstrate that the toxicity increases and the biodegradability is enhanced with the length of the side alkyl chain. The fish immunotoxicity tests indicated that [HMIM]Br has low toxic effects on the stickleback splenocytes by inducing necrosis, likely owing to the bromine anion associated with the imidazolium‐based cation of [HMIM]Br. Recyclability of [HMIM]Br was already examined in our previous study. It was concluded that 75 % of clean IL can be recovered at the end of the treatment and could be used again for similar reactions for several cycles.^10^ All these results contribute to a safe‐by‐design biorefinery involving the studied value chain.

**Table 5:** Overview of the results obtained for [HMIM]Br.

Test	Results	Classification for aquatic environmental hazards
immobilization test (*D. magna*; OECD 202)	EC_50_ 48 h: 414 mg L^−1^; (352–477 mg L^−1^)^[a]^	not classified for acute aquatic hazard
algal growth inhibition test (*P. subcapitata*; OECD 201)	NOEC^[b]^ 72 h: 62.5 mg L^−1^ EC_10_ 72 h: 227 mg L^−1^; (209–242 mg L^−1^) EC_50_ 72 h: 346 mg L^−1^; (327–361 mg L^−1^)	
manometric respirometry test (OECD 301F)	no biodegradation observed	not readily biodegradable; further investigation is needed to conclude on the classification for the long‐term aquatic hazard
fish innate immune responses (*G. aculeatus*)	stimulate immune responses; cytotoxic effect	–^[c]^

[a] 95 % confidence interval. [b] No observed effect concentration. [c] Not relevant.

## Conclusions

The possibility to transfer the methyl imidazolium bromide ([HMIM]Br) treatment to technical lignins was demonstrated, by using alkali grass lignin Protobind 1000 (PB1000) as a commercial reference sample. Safety assessment of [HMIM]Br highlighted the effective flame‐retardant property of this ionic liquid (IL) and provided data on its eco‐toxicological footprint, which does not depart from that of other ILs of the imidazolium family and is useful for future REACH registration. The treatment induced both depolymerization and demethylation of lignin, leading to the formation of additional free phenols. Based on these results and after checking that similar effects were obtained with other commercial technical lignins including Kraft lignin (data not shown), an integrated cascade process combining IL treatment and solvent extractions was designed to optimize the recovery yield of ethyl acetate (EA)‐soluble extracts with enhanced performance compared with the technical lignin. The first step directly provides a functional EA extract, whereas the second and third steps combine extractions with IL treatment to further improve functionalities for applications as antioxidants or building blocks. Indeed, the extracts consisted of free‐phenol‐rich oligomers with antioxidant properties favorably competing with ferulic acid and *tert*‐butylated hydroxytoluene (BHT). Besides their high antiradical activity, these extracts have the advantages of standardized average molar masses (872–937 g mol^−1^), low polydispersity (1.2–1.3), and high free‐phenol content (up to 11.9 mmol g^−1^), which provides opportunities for green biobased innovation in plastics and cosmetics formulations.

## Experimental Section

### General materials and methods

PB1000 was purchased from GreenValue LLC (USA).^27^ [HMIM]Br was synthesized by following a previously reported procedure.^28^ EA was purchased from Carlo Erba Reagents (France) and used as received. All other reagents as well as compounds **A** (ref 410659) and **B** (ref 796883), were purchased from Sigma–Aldrich Chemical Co. (USA) and were used as received.

Thin‐layer chromatography (TLC) experiments were performed with aluminum strips coated with Silica Gel 60 F_254_ from Macherey–Nagel, revealed under UV light (254 nm), then in the presence of a 5 % *w*/*w* ethanolic solution of phosphomolybdic acid. Evaporations were conducted under reduced pressure at temperatures below 35 °C unless otherwise stated. Column chromatography (CC) was performed with an automated flash chromatography PuriFlash system and pre‐packed INTERCHIM PF‐30SI‐HP (30 μm silica gel) columns. ^1^H and ^13^C NMR spectra were recorded in CD_3_OD at 400 or 100 MHz, respectively, with a Bruker Ascend 400 MHz instrument. Chemical shifts are reported in ppm relative to internal references (solvent signal).

### Lignin fractionation and EA solubility tests

**EA extraction (1)**: PB1000 (1 kg) was fractionated by a three‐step sequential solvent extraction process, according to a previously published approach.^8^ The following solvents were used sequentially in a semi‐continuous process: EA, MEK, MEOH. Lignin was loaded in the first solvent in the glass column. After settlement, EA was pumped by HPLC pump at a flow rate of 2–4 mL min^−1^ into the column. The solubilized fraction was collected at different heights in the column. The solvent was removed by vacuum evaporation, and the final solvent was removed by vacuum drying. The recovered solvent was reused in the process. When the concentration of solubilized lignin was very low, the second solvent was added by the pump into the column. For each solvent, the procedure was repeated until, after three solvent extractions, the residual lignin fraction was collected from the column. This fraction was dried at a maximum temperature of 40 °C.

**EA extraction (2)**: PB1000 (6.5 g) was dissolved in EA (250 mL), and the mixture was stirred at room temperature for 30 min. The resulting solid residue was filtered, and the filtrate was recovered. The procedure was repeated nine times to obtain an exhaustive extraction. The combined EA extracts were concentrated under reduced pressure, and the final solvent was removed by vacuum drying. The residual lignin fraction was collected and dried at a maximum temperature of 40 °C.

**EA solubility test**: Lignin solubility was determined gravimetrically by dispersing lignin (100 mg) in EA (10 mL, room temperature, 30 min), then centrifuging the suspension (20 °C, 20 min, 4000 g), and drying the solid residue at 40 °C for 48 h. Solubility was determined in duplicate, based on the amount of solid residue.

### [HMIM]Br treatments

For all treatments, the IL was vacuum‐dried at room temperature before use.

**MW treatment**: MW irradiation experiments were conducted in an Anton Paar Monowave 300 instrument. The sample (200 mg) and [HMIM]Br (2 g) were placed in an Anton Paar 30 mL reaction tube equipped with a magnetic stirrer. The mixture was irradiated with *P*=300 W, *T*_max_=110 °C, ramp 30 s, hold 10 s, with full air cooling and stirring. At the end of the reaction, the solid residue was filtered and washed with water (20 mL) and EA (20 mL). The filtrate was recovered, the layers were separated, and the aqueous layer was extracted with EA (2×20 mL). The combined EA extracts were dried over MgSO_4_ and concentrated under reduced pressure below 35 °C. The crude soluble mixture was analyzed by ^31^P NMR spectroscopy, HPSEC, and thioacidolysis.

**CH treatment**: The sample (200 mg) and [HMIM]Br (2 g) were placed in an Ace pressure tube equipped with a magnetic stirrer under an inert atmosphere, then flushed with Ar. The tube was closed, and the mixture was stirred in an oil bath at 110 °C. After 20 or 40 min, the solid residue was filtered, and the same downstream procedure as for MW irradiation was applied to the solid residue and the filtrate.

### Synthetic procedure for compounds C and D

Acetosyringone (500 mg, 2.5 mmol) and [HMIM]Br (2.5 g, 6 equiv.) were placed in an Anton Paar 30 mL reaction tube equipped with a magnetic stirrer. The mixture was irradiated with *P*=300 W, *T*_max_=110 °C, ramp 30 s, hold 10 s, with full air cooling and stirring. At the end of the reaction, water (20 mL) and EA (20 mL) were added. The layers were separated, and the aqueous layer was extracted with EA (2×20 mL). The combined EA extracts were dried over MgSO_4_ and concentrated under reduced pressure below 35 °C. The crude mixture was purified by flash column chromatography (eluted with 50–100 % EA in cyclohexane) to yield 63 % of product **C** [1‐(3,4‐dihydroxy‐5‐methoxyphenyl)‐ethan‐1‐one] and 26 % of product **D** [1‐(3,4,5‐trihydroxyphenyl)‐ethan‐1‐one]. The products were characterized by NMR spectroscopy (Supporting Information, S4).

Compound **C**: *R*_f_=0.34 (cyclohexane/EA 1:1); ^1^H NMR (400 MHz, CD_3_OD, 25 °C): *δ*_H_=2.53 (s, 3 H, CH_3_), 3.91 (s, 3 H, CH_3_O), 7.19 ppm (m, 2 H, H_2_ and H_6_); ^13^C NMR (100 MHz, CD_3_OD, 25 °C): *δ*_C_=24.9 (CH_3_), 55.3 (CH_3_O), 103.6, 110.2 (C_2_, C_6_), 127.9, 139.9, 144.9, 147.9 (4 C_q_), 198.3 ppm (CO).

Compound **D**: *R*_f_=0.23 (cyclohexane/EA 1:1); ^1^H NMR (400 MHz, CD_3_OD, 25 °C): *δ*_H_=2.48 (s, 3 H, CH_3_), 7.05 (s, 2 H, H_2_ and H_6_); ^13^C NMR (100 MHz, CD_3_OD, 25 °C): *δ*_C_=24.8 (CH_3_), 107.89 (C_2_, C_6_), 128.0, 139.1, 145.2 (4 C_q_), 198.4 ppm (CO).

### Chemical analysis of lignin and lignin‐derived products

**GC–MS analysis**: EA solutions (20 μL, 1 mg mL^−1^) previously dried with Na_2_SO_4_ were silylated with bistrimethylsilyl‐trifluoroacetamide (BSTFA, 100 μL) and GC‐grade pyridine (10 μL). The silylation was completed within a few minutes at room temperature. GC–MS analyses were performed in splitless mode with an Agilent 7890A GC coupled to an Agilent 5977B MS, with a poly(dimethylsiloxane) column (30 m×0.25 mm; Rxi‐5Sil, RESTEK), working in the temperature program mode from 70 to 330 °C at +30 °C min^−1^, over 20 min, with helium as the carrier gas. The chromatographic system was combined with a quadrupole MS operating with electron‐impact ionization (70 eV) and positive‐mode detection, with a source at 230 °C and an interface at 300 °C, and with a 50–800 *m*/*z* scanning range.^29^

**Quantitative** ^**31**^ **P NMR spectroscopy and sample preparation**: Derivatization of the samples with 2‐chloro‐4,4′,5,5′‐tetramethyl‐1,3,2‐dioxaphospholane (TMDP, Sigma–Aldrich, France) was performed according to a reported procedure.^30^ Lignin samples (20 mg) were dissolved in a mixture of anhydrous pyridine and deuterated chloroform (400 μL, 1.6:1 *v*/*v*). Then, a solution (150 μL) containing cyclohexanol (6 mg mL^−1^) and chromium(III) acetylacetonate (3.6 mg mL^−1^), which served as internal standard and relaxation reagent, respectively, and TMDP (75 μL) were added. NMR spectra were acquired without proton decoupling in CDCl_3_ at 162 MHz, with a Bruker Ascend 400 MHz spectrometer. A total of 128 scans were acquired with a delay time of 6 s between two successive pulses. The spectra were processed by using Topspin 3.1. All chemical shifts were reported in ppm relative to the product of phosphorylated cyclohexanol (internal standard), which has been observed to give a doublet at 145.1 ppm. The content in hydroxyl groups (in mmol g^−1^) was calculated on the basis of the integration of the phosphorylated cyclohexanol signal and by integration of the following spectral regions: aliphatic hydroxyls (150.8–146.4 ppm), condensed phenolic units (145.8–143.8 ppm; 142.2–140.2 ppm), syringyl phenolic hydroxyls (143.8–142.2 ppm), guaiacyl phenolic hydroxyls (140.2–138.2), *p*‐hydroxyphenyl phenolic hydroxyls (138.2–137.0 ppm) and carboxylic acids (136.6–133.6 ppm).

**Thioacidolysis**: Thioacidolysis of lignins (5 mg) was performed according to a literature protocol,^31^ by using heneicosane (C_21_H_44_, Fluka) as internal standard. Lignin‐derived *p*‐hydroxyphenyl (H), guaiacyl (G) and syringyl (S) thioacidolysis monomers were analyzed as their trimethylsilyl derivatives by GC–MS (Saturn 2100, Varian) equipped with a poly(dimethylsiloxane) column (30 m×0.25 mm; SPB‐1, Supelco) and by using the following heating program: 40–180 °C at 30 °C min^−1^, then 180–260 °C at 2 °C min^−1^. The MS was an ion trap with an ionization energy of 70 eV and positive‐mode detection. The determination of the thioethylated H, G, and S monomers was performed from ion chromatograms reconstructed at *m*/*z*=239, 269 and 299, respectively, compared with the internal standard signal measured from the ion chromatogram reconstructed at *m*/*z*=(57+71+85). The molar yield of the detected thioethylated monomers was calculated on the basis of the Klason lignin content of the sample, determined according to a published procedure.^32^

**Molar mass distribution**: *M*_n_ and *M*_w_ of the samples were estimated by HPSEC using a styrene–divinylbenzene PL‐gel column (Polymer Laboratories, 5 μm, 100 Å, 600 mm×7.5 mm inner diameter) with a photodiode array detector (Dionex Ultimate 3000 UV/Vis detector) set at 280 nm, and by using BHT‐stabilized THF (1 mL min^−1^) as eluent. The samples were solubilized in THF and filtered through a polytetrafluoroethylene membrane (0.45 μm) before injection. The molar mass averages were assessed from the apparent molar masses determined by a calibration curve based on polyethylene oxide standards (Igepal, Aldrich) and lignin model dimers.^33^

### Assessment of antioxidant properties

**Preparation of the solutions**: A lignin sample was weighed into a 2 mL microfuge tube, and the solvent (90:10 *v*/*v* dioxane/water mixture) was added to obtain concentrations between 0.1 and 0.5 mg mL^−1^. The dispersion was homogenized by using a vortex (Heidolph TOP‐MIX 94323, Fisher Scientific Bioblock, Vaulx Milieu, France) for 30 s at 20 000 Hz. The resulting solutions were tested for their radical‐scavenging activity.

**Measurement of the free‐radical‐scavenging activity by DPPH^.^ test**: The free‐radical‐scavenging activity of the samples was evaluated by measuring their reactivity toward the stable free radical DPPH^.^ according to a published method.^14^ In a quartz cuvette, the sample dioxane/water solution (77 μL) was added to 3 mL of a 6×10^−5^ mol L^−1^ DPPH^.^ solution, prepared daily in absolute ethanol. The absorbance at 515 nm of each sample was monitored by using an UV/Visible double‐beam spectrophotometer (Shigematsu Scientific Instrument, USA), until reaching a plateau. A blank was prepared under the same conditions, by using 77 μL of the solvent instead of the sample solution. All kinetics were obtained from at least six solutions, prepared from three different lignin preparations. The kinetics of the disappearance of DPPH^.^ were obtained by calculating at each time the difference between the absorbance of the blank solution and the absorbance of the sample. When the absorbance reached a plateau, the percentage of residual DPPH^.^ was calculated and plotted versus the concentration of soluble lignin in the sample tested. The concentration of antioxidant extract needed to reduce 50 % of the initial DPPH^.^ (EC_50_, with EC standing for efficient concentration) was determined from this linear curve.

### Safety assessment of [HMIM]Br

The overall multicriteria safety analysis has been inspired by the global strategy developed by some of the authors of this manuscript for greener use of ILs in general, as exemplified by Eshetu et al.^34^ in the case of energy storage in electrochemical devices.

**Fire hazard**: Examined by fire calorimetry testing based on the use of the most polyvalent fire calorimeter designated as the Fire Propagation Apparatus, following ISO 12136. See also the Supporting Information for further details on procedures and complementary details on achieved experimental data.

**Corrosivity screening tests**: Carbon and stainless‐steel specimens, partially immersed in plastic cells containing neat IL and IL added with 10 % water, following a home‐made procedure (exposure in an oven regulated at 100 °C for 8 days), with mass loss determined before and after exposure with a calibrated balance, inspired by the procedure developed for IL corrosivity assessment by German ILs producer IO‐LI‐TEC.

**Ecotoxicity tests**: Ecotoxicity tests required by annex VII of the REACH regulation (substance manufactured or imported into the European Union in quantities between 1 and 10 tons per year) have been performed. They are presented briefly in Table 6. In addition to these regulatory tests, fish immunomarker tests were conducted to study possible long‐term effects on aquatic ecosystems. The protocol is detailed in a previous paper.^26^

**Table 6:** Summary of the ecotoxicity tests performed for [HMIM]Br.

Organisms	Test method	Effect	Endpoints	Expression of results	Test duration
micro‐algae, *P. subcapitata*	OECD 201, 2011	chronic	growth	NOEC; EC_10_; EC_50_	72 h
micro‐crustaceans, *D. magna*	OECD 202, 2004	acute	mobility	EC_50_	48 h
activated sludge receiving predominantly domestic sewage	OECD 301F, 1992	ready biodegradability	oxygen consumption	% biodegradation	28 days

## Conflict of interest

*The authors declare no conflict of interest*.

## Supporting information

As a service to our authors and readers, this journal provides supporting information supplied by the authors. Such materials are peer reviewed and may be re‐organized for online delivery, but are not copy‐edited or typeset. Technical support issues arising from supporting information (other than missing files) should be addressed to the authors.

Supplementary
