# Supplementary material for: Enhancing the Antioxidant Activity of Technical Lignins by Combining Solvent Fractionation and Ionic‐Liquid Treatment
Source: ChemSusChem. 2019 Sep 24;12(21):4799–809. doi: 10.1002/cssc.201901916 (PMC6899661; doi:10.1002/cssc.201901916)
Supplement: Supplementary file 1 — Supplementary [file CSSC-12-4799-s001.pdf]

CHEMISTRY & SUSTAINABILITY
CHEMSUSCHEM
ENERGY & MATERIALS

Supporting Information

Enhancing the Antioxidant Activity of Technical Lignins by Combining Solvent Fractionation and Ionic-Liquid Treatment

Amel Majira,[a] Blandine Godon,[b] Laurence Foulon,[b] Jacinta C. van der Putten,[c] Laurent Cézard,[a] Marina Thierry,[a] Florian Pion,[a] Anne Bado-Nilles,[d] Pascal Pandard,[d] Thangavelu Jayabalan,[d] Véronique Aguié-Béghin,[b] Paul-Henri Ducrot,[a] Catherine Lapierre,[a] Guy Marlair,[d] Richard J. A. Gosselink,[c] Stephanie Baumberger,*[a] and Betty Cottyn*[a]

cssc_201901916_sm_miscellaneous_information.pdf

Author Contributions

A. Majira synthesised the model compounds, performed the ^{31}P NMR analyses of the raw and derivated lignin samples and provided technical support for the experiments with [HMIM]Br. L. Cézard performed the thioacidolysis analysis of the raw and derivatized lignin samples. M. Thierry contributed to the optimization of the IL treatment and investigated the effect of the treatment on PB1000, in the framework of her MSc thesis. F. Pion prepared PB1000 fraction F4 at the lab scale and contributed to the design of the integrated cascade process. A. Bado-Nilles, P. Pandard, T. Jayabalan and G. Marlair performed the safety related analysis of [HMIM]Br and contributed to the selection of process conditions. P.-H. Ducrot contributed to the design of the IL route for lignin conversion and to the elucidation of reaction mechanisms. C. Lapierre performed the analysis of lignin extractives and contributed to the identification of lignin demethylation and depolymerization products by mass spectrometry. B. Godon, L. Foulon and V. Aguié-Béghin conceived, performed and analyzed the measurement of antioxidant activity of the raw and derivatized lignin samples. J.C. van der Putten performed the semi-continuous fractionation processes and characterized lignin fractions F1–F3. R.J.A. Gosselink coordinated the semi-continuous fractionation process implemented on PB1000 and provided the characterized lignin fractions F1–F3. S. Baumberger coordinated the study within the framework of the Zelcor project and designed the cascade process. B. Cottyn designed the [HMIM]Br process, implemented it to PB1000 and its fractions and coordinated the analysis of the lignin raw material and derivatives. All the authors participated in the scientific discussions, read, and approved the final manuscript.

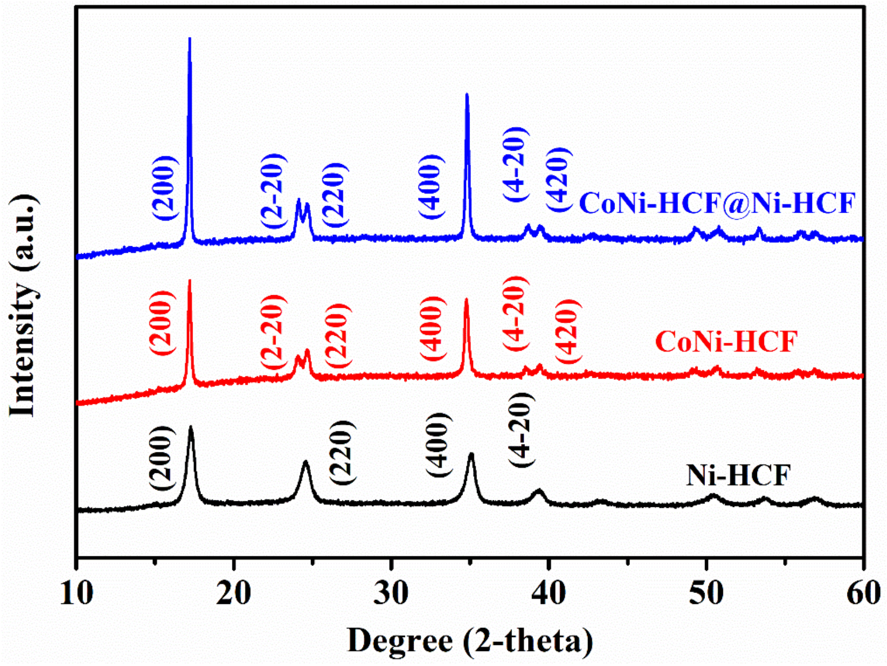

Figure S1. XRD pattern of Ni-HCF, CoNi-HCF and CoNi-HCF@Ni-HCF, respectively.

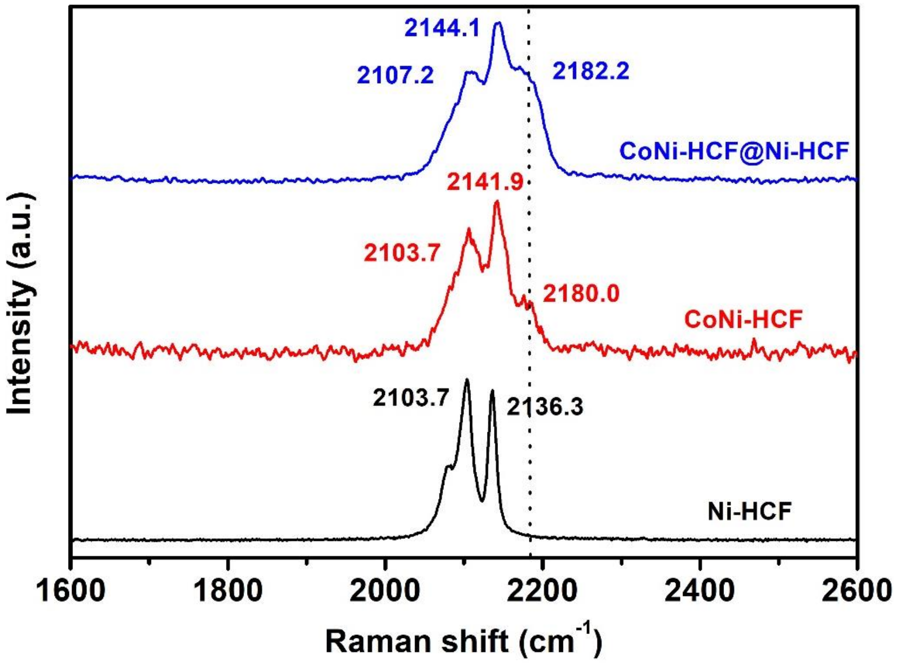

Figure S2. Raman spectra of Ni-HCF, CoNi-HCF and CoNi-HCF@Ni-HCF, respectively.

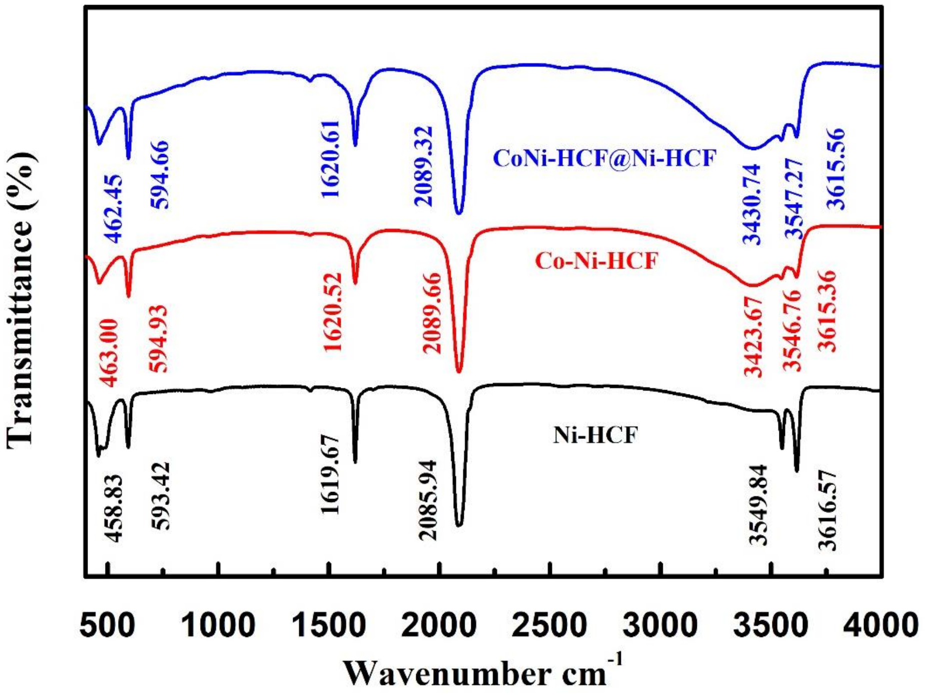

Figure S3. Fourier Transform Infrared Spectroscopy (FTIR) pattern of Ni-HCF, CoNi-HCF and CoNi-HCF@Ni-HCF, respectively.

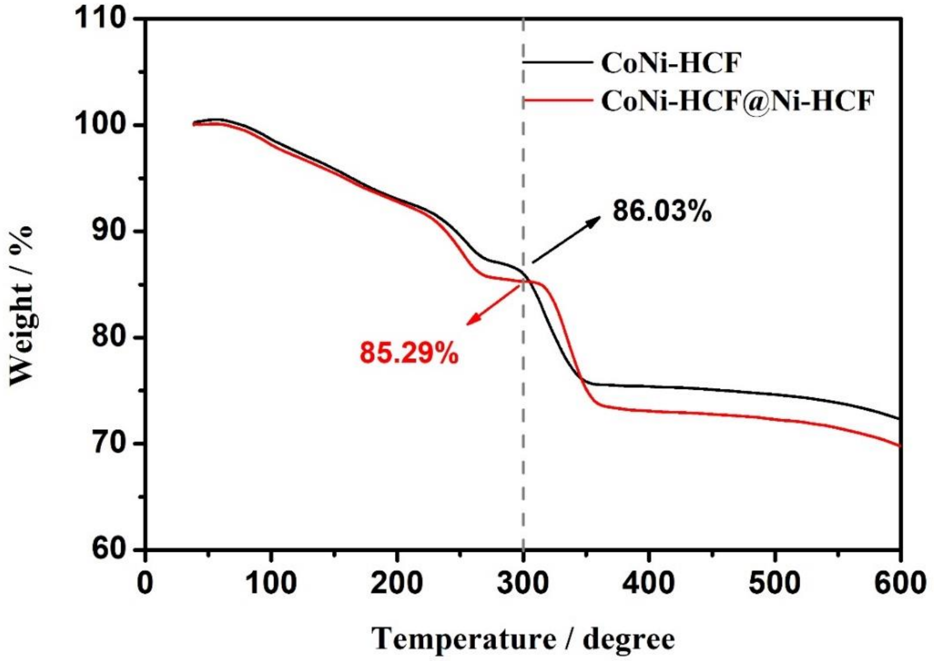

Figure S4. TG curves of CoNi-HCF and CoNi-HCF@Ni-HCF.

Table S1. The ICP-OES result of the Fe content in the electrolyte

Sample Name	Fe Content
Ni-HCF	0.017 mg L^{-1}
CoNi-HCF@Ni-HCF	0.031 mg L^{-1}
CoNi-HCF	0.053 mg L^{-1}

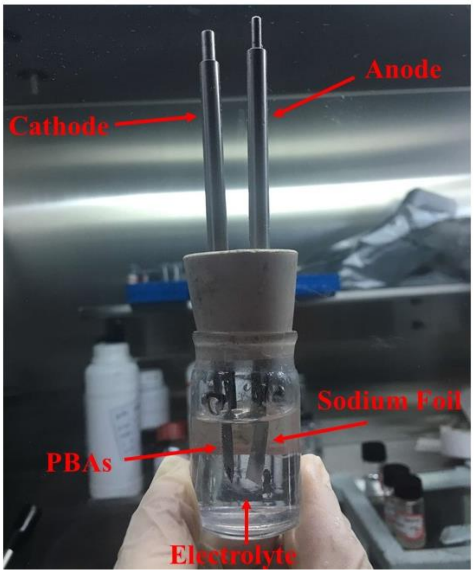

Figure S5. Photograph of the home-made half-cell. The homemade half-cell was assembled by using Ni-HCF, CoNi-HCF@Ni-HCF or CoNi-HCF as working electrode, sodium metal as counter electrode and the electrolyte was 1.0 mol L^{-1} $NaClO_4$, in EC/DEC (1:1 vol) solution with 2 wt% FEC was solvent. After 500 galvanostatic charge-discharge cycles at 300 mA g^{-1} in the glovebox with H_2O and O_2 content less than 10 ppm, the content of Fe in the electrolyte solution was analyzed by inductively coupled plasma emission spectrometer (ICP-OES).

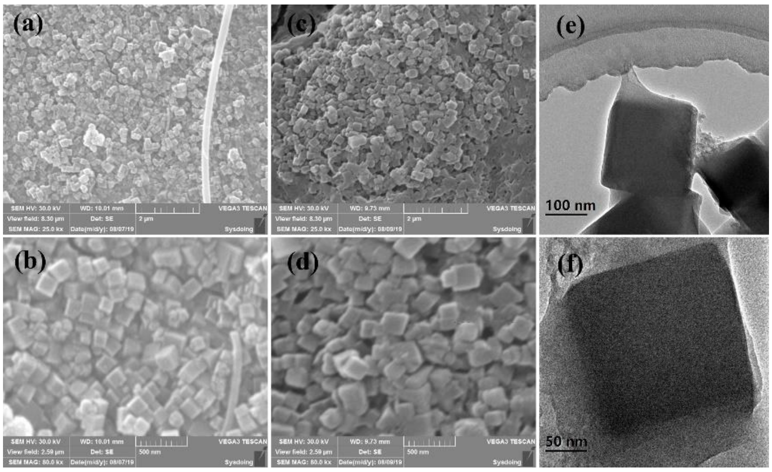

Figure S6. The morphology of the CoNi-HCF@Ni-HCF at fresh state and after 1000 cycles. (a,b) the SEM patterns at fresh state. (c,d) the SEM patterns after cycling. (e,f) the TEM patterns after cycling.

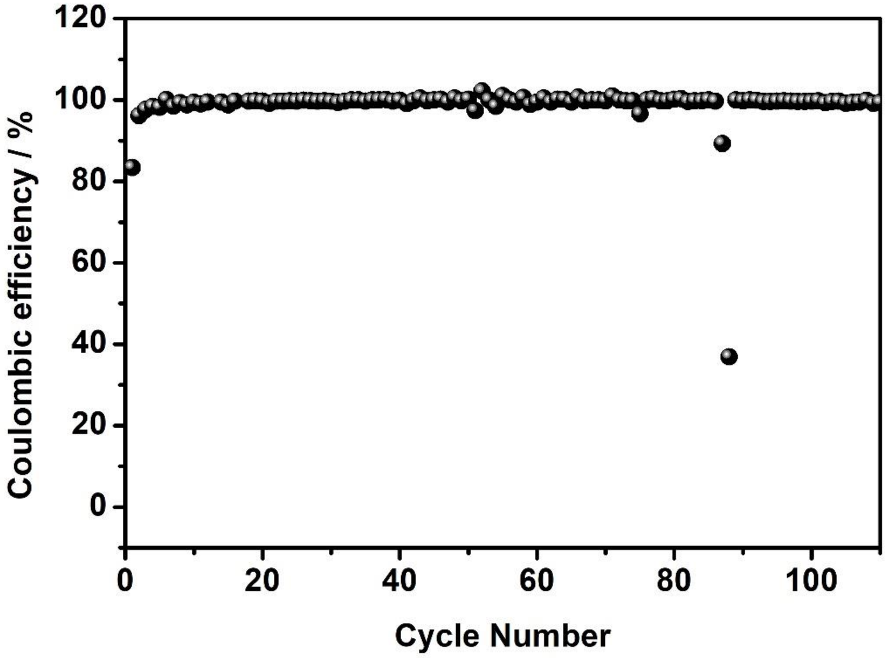

Figure S7. The coulombic efficiency of CoNi-HCF@Ni-HCF during the rate testing.

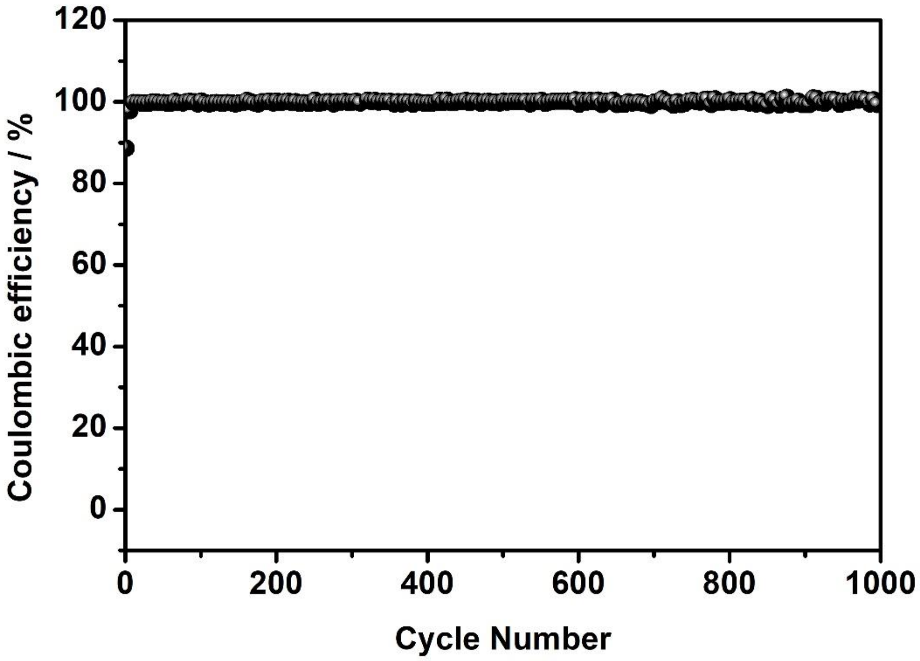

Figure S8. The coulombic efficiency of CoNi-HCF@Ni-HCF during the cycling testing.

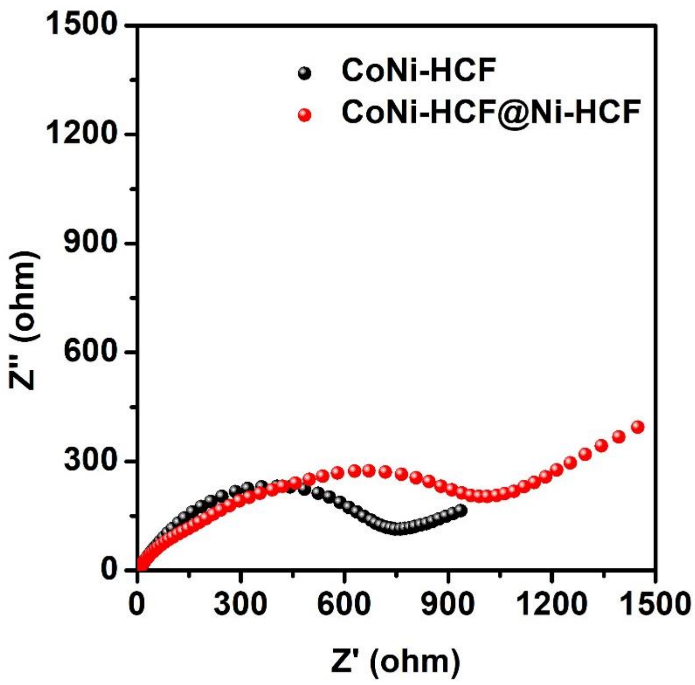

Figure S9. The EIS curves of CoNi-HCF and CoNi-HCF@Ni-HCF.

Table S2. The electrochemical performances of different PBAs

Materials	Capacity	Rate performance	Cycle stability
$Na_{0.84}Ni[Fe(CN)_6]_{0.71}$[1]	66 mA h g^{-1} at 20 mA g^{-1}	--	99.7% after 200 cycles at 20 mA g^{-1}
$FeFe(CN)_{6}$[2]	120 mA h g^{-1} at 60mA g^{-1}	78 mA h g^{-1} at 20 C	87% after 500 cycles at 2C
$Na_{0.61}Fe[Fe(CN)_6]_{0.94}\square_{0.06}$[3]	170mA h g^{-1} at 25mA g^{-1}	70 mA h g^{-1} at 600 mA g^{-1}	100% after 150 cycles at 0.2C
$Na_2Zn_3[Fe(CN)_6]_2$[4]	56.4 mA h g^{-1} at 10mA g^{-1}	--	85% after 50 cycles at 10 mA g^{-1}
$Cu_3[Fe(CN)_6]_2$[5]	44 mA h g^{-1} at 20mA g^{-1}	25 mA h g^{-1} at 100 mA g^{-1}	57% after 50 cycles at 20 mA g^{-1}
$Na_{1.56}FeFe(CN)_6$[6]	103.6 mA h g^{-1} at 20 mA g^{-1}	90 mA h g^{-1} at 100 mA g^{-1}	97% after 400 cycles at 20 mA g^{-1}
$Na_{1.63}Fe[Fe(CN)_6]_{0.89}$[7]	150 mA h g^{-1} at 25 mA g^{-1}	--	90% after 200 cycles at 25 mA g^{-1}
$Na_{1.73}Fe[Fe(CN)_6]_{0.98}$[8]	156 mA h g^{-1} at 5 mA g^{-1}	96.8 mA h g^{-1} at 100 mA g^{-1}	61.3% after 1000 cycles at 100 mA g^{-1}
$Na_{1.92}FeFe(CN)_6$[9]	160 mA h g^{-1} at 10 mA g^{-1}	145 mA h g^{-1} at 10 C	80% after 750 cycles at 2 C
$Na_{1.32}Mn[Fe(CN)_6]_{0.83}$[10]	109 mA h g^{-1} at 50 mA g^{-1}	< 40 mA h g^{-1} at 20 C	90% after 100 cycles at 0.5 C
$Na_{1.72}Mn[Fe(CN)_6]_{0.99}$[11]	134mA h g^{-1} at 6 mA g^{-1}	45 mA h g^{-1} at 40 C	90% after 30 cycles at 6 mA h g^{-1}
$Na_{1.89}Mn[Fe(CN)_6]_{0.97}$[12]	150 mA h g^{-1} at 15 mA g^{-1}	120 mA h g^{-1} at 20 C	75% after 500 cycles at 105 mA h g^{-1}
$Na_{1.96}Mn[Mn(CN)_6]_{0.99}$[13]	209 mA h g^{-1} at 40 mA g^{-1}	157 mA h g^{-1} at 5 C	75% after 100 cycles at 2C
$Na_{1.60}Co[Fe(CN)_6]_{0.90}$[14]	139 mA h g^{-1} at 70 mA g^{-1}	121 mA h g^{-1} at 60 C	71% after 100 cycles at 0.6 C
$Na_{1.85}Co[Fe(CN)_6]_{0.99}$[15]	153 mA h g^{-1} at 10 mA g^{-1}	60 mA h g^{-1} at 500 mA g^{-1}	90% after 200 cycles at 200 mA h g^{-1}
Fe-HCF NSs@ GRs[16]	110 mA h g^{-1} at 150 mA g^{-1}	95 mA h g^{-1} at 1500 mA g^{-1}	90% after 500 cycles at 1 C
$Na_{0.53}Ce[Fe(CN)_6]_{0.77}$[17]	57.9 mA h g^{-1} at 20 mA g^{-1}	52.7 mA h g^{-1} at 150	77% after 80 cycles at

		mA g^{-1}	2.5 C
$Na_{1.68}Ni_{0.14}Co_{0.86}[Fe(CN)_6]_{0.84}$[18]	110 mA h g^{-1} at 750 mA g^{-1}	111 mA h g^{-1} at 300 mA g^{-1}	83% after 600 cycles at 750 mA h g^{-1}
This work	98.7 mA h g^{-1} at 300 mA g^{-1}	79.1 mA h g^{-1} at 1500 mA g^{-1}	88% after 1000 cycles at 300 mA h g^{-1}

[1] Y. You, X.-L. Wu, Y.-X. Yin, Y.-G. Guo, Journal of Materials Chemistry A, 1 (2013) 14061-14065.
[2] X. Wu, W. Deng, J. Qian, Y. Cao, X. Ai, H. Yang, Journal of Materials Chemistry A, 1 (2013) 10130-10134.
[3] Y. You, X.-L. Wu, Y.-X. Yin, Y.-G. Guo, Energy & Environmental Science, 7 (2014) 1643-1647.
[4] H. Lee, Y.-I. Kim, J.-K. Park, J.W. Choi, Chemical communications, 48 (2012) 8416-8418.
[5] S. Jiao, J. Tuo, H. Xie, Z. Cai, S. Wang, J. Zhu, Materials Research Bulletin, 86 (2017) 194-200.
[6] W.-J. Li, S.-L. Chou, J.-Z. Wang, Y.-M. Kang, J.-L. Wang, Y. Liu, Q.-F. Gu, H.-K. Liu, S.-X. Dou, Chemistry of Materials, 27 (2015) 1997-2003.
[7] Y. You, X. Yu, Y. Yin, K.-W. Nam, Y.-G. Guo, Nano research, 8 (2015) 117-128.
[8] W. Tang, Y. Xie, F. Peng, Y. Yang, F. Feng, X.-Z. Liao, Y.-S. He, Z.-F. Ma, Z. Chen, Y. Ren, Journal of The Electrochemical Society, 165 (2018) A3910-A3917.
[9] L. Wang, J. Song, R. Qiao, L.A. Wray, M.A. Hossain, Y.-D. Chuang, W. Yang, Y. Lu, D. Evans, J.-J. Lee, Journal of the American Chemical Society, 137 (2015) 2548-2554.
[10] T. Matsuda, M. Takachi, Y. Moritomo, Chemical Communications, 49 (2013) 2750-2752.
[11] L. Wang, Y. Lu, J. Liu, M. Xu, J. Cheng, D. Zhang, J.B. Goodenough, Angewandte chemie international edition, 52 (2013) 1964-1967.
[12] J. Song, L. Wang, Y. Lu, J. Liu, B. Guo, P. Xiao, J.-J. Lee, X.-Q. Yang, G. Henkelman, J.B. Goodenough, Journal of the American Chemical Society, 137 (2015) 2658-2664.
[13] H.-W. Lee, R.Y. Wang, M. Pasta, S.W. Lee, N. Liu, Y. Cui, Nature communications, 5 (2014) 5280.
[14] M. Takachi, T. Matsuda, Y. Moritomo, Applied Physics Express, 6 (2013) 025802.
[15] X. Wu, C. Wu, C. Wei, L. Hu, J. Qian, Y. Cao, X. Ai, J. Wang, H. Yang, ACS applied materials & interfaces, 8 (2016) 5393-5399.
[16] J. Luo, S. Sun, J. Peng, B. Liu, Y. Huang, K. Wang, Q. Zhang, Y. Li, Y. Jin, Y. Liu, ACS applied materials & interfaces, 9 (2017) 25317-25322.
[17] J. Peng, C. Li, J. Yin, J. Wang, Y. Yu, Y. Shen, J. Fang, A. Chen, Y. Xu, R. Rehman, ACS Applied Energy Materials, 2 (2018) 187-191.
[18] J. Peng, J. Wang, H. Yi, W. Hu, Y. Yu, J. Yin, Y. Shen, Y. Liu, J. Luo, Y. Xu, Advanced Energy Materials, 8 (2018) 1702856.
